# Maximizing Relayed ^1^H Hyperpolarization Transfer by Slow-Fast MAS NMR Spectroscopy

**DOI:** 10.1021/acs.jpca.4c02452

**Published:** 2024-08-09

**Authors:** Saumya Badoni, Pierrick Berruyer, Lorenzo Niccoli, Anne Lesage, Lyndon Emsley

**Affiliations:** † Institut des Sciences et Ingénierie Chimiques, Ecole Polytechnique Fédérale de Lausanne (EPFL), CH-1015 Lausanne, Switzerland; ‡ Université de Lyon, Centre de Resonance Magnétique Nucléaire (CRMN) à Très Hauts Champs de Lyon (UMR 5082 - CNRS, ENS Lyon, UCB Lyon 1), 69100 Villeurbanne, France; § Center of Magnetic Resonance (CERM), University of Florence, 50019 Sesto Fiorentino, Italy; ∥ Departement of Chemistry “Ugo Schiff”, University of Florence, 50019 Sesto Fiorentino, Italy; ⊥ Consorzio Interuniversitaio Risonanze Magnetiche Metalloproteine (CIRMMP), 50019 Sesto Fiorentino, Italy

## Abstract

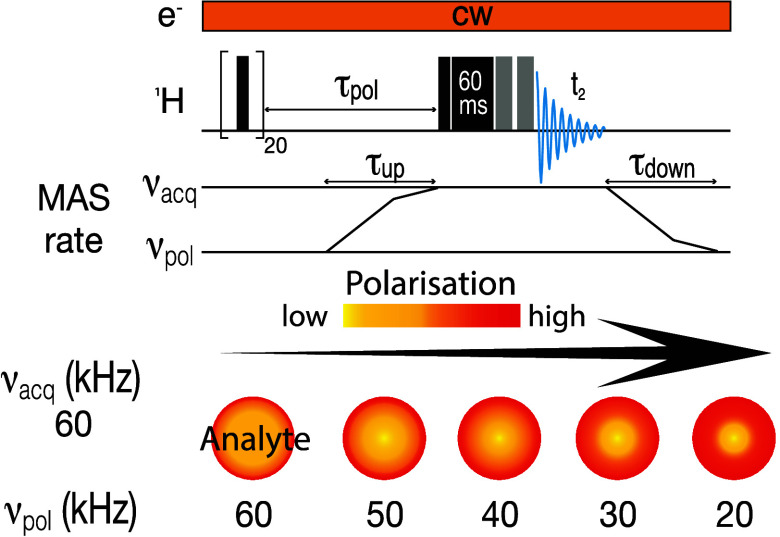

^1^H-detected dynamic nuclear polarization (DNP)-enhanced fast magic angle spinning (MAS) NMR experiments provide unprecedented sensitivity to study the structure and dynamics in advanced materials and biomolecules. However, in relayed DNP experiments, DNP enhancements decrease with faster MAS rates, which is detrimental for sensitivity. The decrease is because ^1^H-^1^H spin diffusion rates are significantly reduced at fast MAS frequencies. To improve sensitivity at these fast MAS rates, here, we propose to combine fast polarization build-up by relay at slow MAS rate with high-resolution ^1^H NMR at fast MAS for acquisition. We perform experiments on l-histidine·HCl·H_2_O with MAS rates of up to 65 kHz using a 0.7 mm DNP probe at 18.8 T and 100 K. We obtain a 35% improvement in sensitivity in experiments where the sample is polarized at 20 kHz MAS and where the signal is acquired at 60 kHz MAS.

## Introduction

High field NMR spectroscopy combined with fast magic angle spinning (MAS) has found extensive application for the characterization of solid materials, ranging from biomolecules and pharmaceutical formulations to inorganic materials.^1^ The advent of dynamic nuclear polarization (DNP) under MAS^2,3^ provides significant sensitivity enhancement, and its integration with high-fields and fast MAS has broadened the scope of application to highly sensitive and well-resolved ^1^H based detection schemes.^4^

MAS DNP of materials is today most commonly performed by impregnation, which involves wetting the analyte with a radical containing solution.^5,6^ Subsequent irradiation of the sample with microwaves at or near the electron paramagnetic resonance (EPR) frequency of the radical, leads to hyperpolarization on the ^1^H nuclei near the radicals, which then diffuses to the bulk of the radical-free material by spontaneous ^1^H-^1^H spin diffusion. This approach, denoted relayed DNP, has been applied to many substrates, and can provide very significant sensitivity gains.^7−11^

In reference,^12^ the authors have shown how this approach can be used at MAS rates up to 65 kHz and combined with ^1^H detection.^12,13^ In this regime, it was found that there is a balance in overall sensitivity due to the competing effects of the reduction in line-width along with reduction in spinning sideband intensity, leading to more intense signals, and reduction in the spin-diffusion coefficient, *D*, as spin rates are increased, leading to less hyperpolarization. In the case of an impregnated powder of l-histidine·HCl·H_2_O, spinning rates of between 40-50 kHz provided optimum sensitivity, with sensitivity falling off at higher MAS rates.^13^

Indeed, for analytes with particle sizes *L* in the range of  or larger, (where *T*_1_ is the longitudinal relaxation time of the analyte) the hyperpolarization process is diffusion limited.^14−16^ The quantity of polarization relayed into the analyte depends on the spin-diffusion coefficient. ^1^H spin-diffusion in organic solids is a coherent process typically driven by ^1^H homonuclear dipolar couplings, and occurs via quantum-mechanical flip-flop processes in the coupled ^1^H network.^17,18^ Faster MAS leads to better averaging of the ^1^H dipolar couplings and the spin-diffusion coefficient typically has an inverse dependence on the MAS rate.^12,19,20^

To avoid the drawback of slower spin diffusion at fast MAS rates, and to improve sensitivity for fast MAS ^1^H spectra under relayed DNP conditions, here we propose to combine slow MAS rates, where strong ^1^H coupling allows for faster spin diffusion, with fast MAS rates which enable high-resolution ^1^H NMR spectroscopy. We thus perform experiments with polarization build-up at slow MAS rates, and then acquire spectra at fast MAS. We demonstrate the method on l-histidine·HCl·H_2_O, where we find that for MAS spectra acquired at 60 kHz, sensitivity is improved 35% when the sample is polarized at 20 kHz MAS compared to a sample polarized at 60 kHz.

We note that a similar method has previously been used to enhance hyperpolarization in pulse-cooling experiments for dilute spins, and demonstrated for powdered SnO_2_ particles at MAS rates up to 12.5 kHz on a 3.2 mm DNP probe, to pump polarization via ^119^Sn spin diffusion.^21^ While for such dilute spins, the dipolar couplings are nearly fully averaged at that relatively slow MAS rate, the much stronger ^1^H dipolar couplings are still active up to very fast MAS rates. Changing the spinning speed during an experiment has also been proposed previously for correlating static patterns to isotropic chemical shifts,^22^ and for broadening CP matching conditions.^23^ It has also been used in the context of DNP, but in the opposite sense, polarizing with a spinning sample and detecting on a static sample for broadband quadrupolar nuclei.^24^

## Experimental Section

l-histidine·HCl·H_2_O was obtained from Sigma-Aldrich, and then crushed with a mortar and pestle for 30 minutes to reduce particle sizes. Subsequently, the sample was impregnated with 32 mM HyTEK-2, the best performing radical to date under these experimental conditions,^25,26^ in 1,1,2,2-tetrachloroethane (TCE)^27^ and packed into a 0.7 mm zirconia rotor.

Experiments were performed on an 800 MHz (18.8 T) magnet equipped with a 9.7 T 527 GHz gyrotron microwave source. The NMR magnetic field was swept so as to obtain maximum enhancement for HyTEK-2. A 0.7 mm low-temperature DNP MAS probe was used in ^1^H-^13^C-^15^N triple mode configuration. Samples were stably spun between 20-60 kHz for the experiments. Before measurements, four freeze-thaw cycles were performed on the sample for degassing.^28^

The DEPTH^29,30^ pulse sequence was used for background suppression to record ^1^H MAS NMR spectra, due to the large ^1^H probe background signal. The two 180° pulses before detection are phase cycled according to the Exorcycle^31^ scheme and require 16 scans for complete phase cycling and background suppression. However, for spectra with high signal-to-noise ratio, 8 scans were sufficient for background suppression and to obtain a flat baseline (Figure S1). To record ^1^H NMR spectra of l-Histidine·HCl·H_2_O impregnated with 32 mM HyTEK-2 in TCE, spin-locks of 130, 150, 150, 140, and 130 kHz were used after optimization for MAS rates of 20, 30, 40, 50, and 60 kHz, respectively, for TCE signal suppression (Figure S2).^32^ Subsequently, the ^1^H build-up time for the l-histidine·HCl·H_2_O signals under μwave irradiation was recorded using the saturation recovery experiment, using ^13^C cross-polarization (CP) MAS^33,34^ NMR (at 30, 40, and 50 kHz MAS rates) and ^1^H DEPTH NMR with spin-lock pulse sequences (at 20 and 60 kHz MAS rates).

We note that for the experiments where the MAS rate is changed, the MAS rate change profiles are reproducible with respect to the spin-up and spin-down cycles (the recorded MAS rate over the period of the experiments is provided as Supporting Information (SI) Data) and the rate change profile for ν_pol_ = 20 kHz and ν_acq_ = 30, 40, 50, 60 kHz, and ν_pol_ = 20, 30, 40, 50 kHz and ν_acq_ = 60 kHz, is given in the SI, Figure S3.

**Figure 1:**
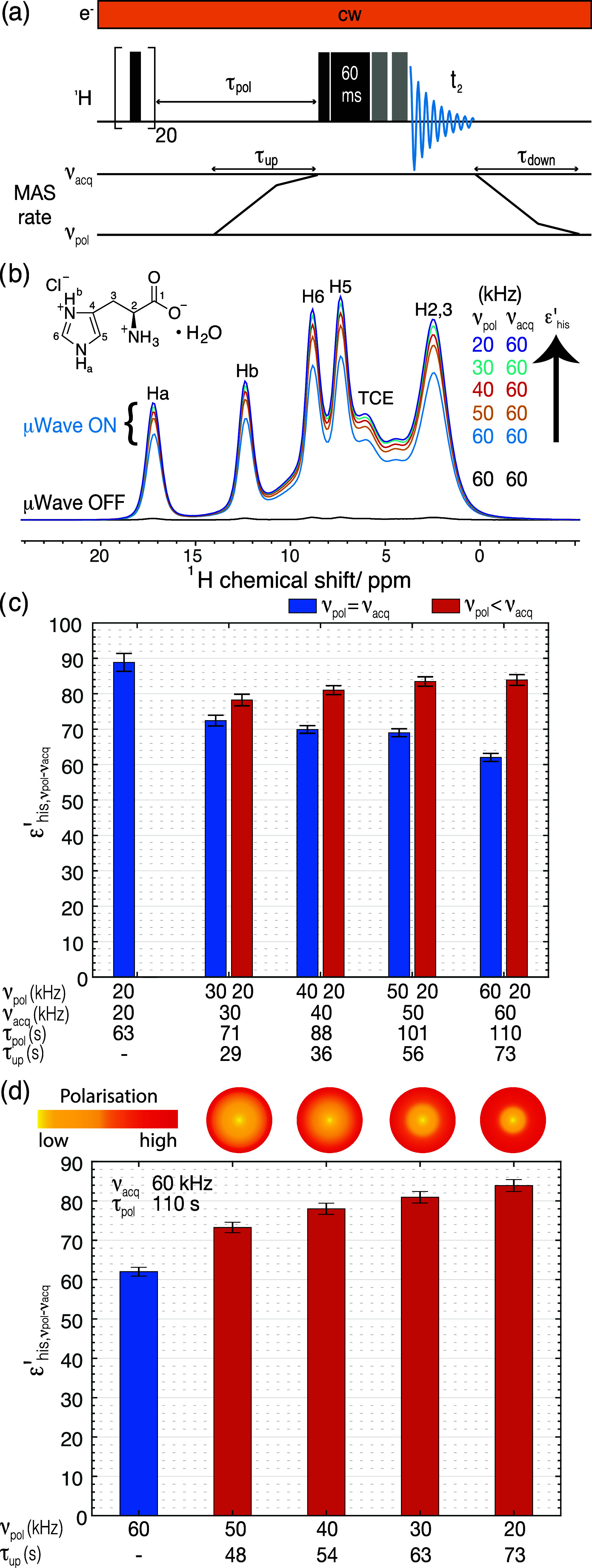
(a) Pulse sequence for the slow-fast MAS experiments, shown with spin-lock and DEPTH pulses before detection as discussed in the text. The solid black bars represent 90° pulses, and gray bars represent 180° pulses. (b) ^1^H DNP MAS NMR spectra recorded with ν_acq_ of 60 kHz and ν_pol_ of 20, 30, 40, and 50 kHz under microwave irradiation, and a standard experiment at 60 kHz with and without microwave irradiation. (c) and (d) show ε′_his,νpol-νacq_ and corresponding error bars, measured for the standard experiments (blue bars), and slow-fast MAS experiments (red bars) at (c) ν_pol_ = 20 kHz and ν_acq_ = 20, 30, 40, 50, 60 kHz, and (d) ν_pol_ = 20, 30, 40, 50, 60 kHz and ν_acq_ = 60 kHz, at τ_pol_ = *T*_opt,his_. Above the bar graphs shown in (d), the spheres give a schematic representation of the relative amount of polarization inside the histidine particles at τ_pol_ = *T*_opt,his_ = 110 s for the different experiments with ν_acq_ = 60 kHz. In (c) and (d) the corresponding ν_pol_, ν_acq_, τ_pol_, and τ_up_ values used for the experiments are given with the bar graphs. The error bars were measured with the formula given in eq S1, SI.

The slow-fast MAS experiment, depicted in Figure 1a, involves saturation pulses, followed by polarization build-up at MAS rate *ν*_pol_ for a time (*T*_opt_–τ_up_), and then a MAS rate change to *ν*_acq_ during time *τ*_up_, followed by a 90° pulse to create transverse magnetization, and a spinlock and two 180° pulses before acquisition, i.e the solvent suppression and the DEPTH pulse sequence elements, respectively. Here, *τ*_up_ is the time it takes to switch the MAS rate from *ν*_pol_ to *ν*_acq_, such that *ν*_pol_ < *ν*_acq_. Before the next scan, the MAS was switched back to the MAS rate *ν*_pol_ during *τ*_down_, as shown in Figure 1a. To set up the slow-fast MAS pulse sequence in TopSpin, TopSpin python MACROS were used so as to set up the experiment as different blocks involving saturation pulses, a delay for polarization build-up, commands for the MAS3 unit to change the MAS rate from *ν*_pol_ to *ν*_acq_, pulses for acquisition, followed by commands for MAS3 to change the MAS rate from *ν*_acq_ to *ν*_pol_. Subsequently, the next scan was recorded. The MACROS are given in SI. In this manner, each scan was recorded as a separate experiment number, and after completion of phase cycling (here 8 scans), these scans were added together to provide the final spectrum.

The slow-fast MAS experiments were performed with a minimum ν_pol_ of 20 kHz to avoid MAS instability in the 0.7 mm probe. Furthermore, the target MAS rate in these experiments was set to be 0.5-1 kHz higher than the desired MAS rate, ν_acq_, to reduce the duration of τ_up_, as the MAS3 rate change profile used herein slows down the rate of MAS change close to the target MAS rate set-point (see Figure S3).

DNP enhancements were determined by computing the ratio of the integrated ^1^H signal of the secondary amine at 17 ppm, with and without μwave irradiation. For experiments with a MAS rate change, the enhancement ε′_νpol-νacq_, is the ratio of signal integrals obtained from spectra recorded using a MAS rate change under microwave irradiation, and a standard experiment without microwave irradiation, while ε_νpol-νacq_, represents the ratio of signal integrals obtained from spectra recorded using a MAS rate change with and without microwave irradiation. Experimentally, these were determined by taking the ratio of the ^1^H signal integral for 30, 40, 50, and 60 kHz experiments and ^1^H signal intensity for the 20 kHz experiments (due to minor sideband overlap at the downfield edge of peak Ha in the μwave on experiment at 20 kHz) of the secondary amine at 17 ppm. The effect of temperature variation from frictional heating due to MAS rate changes on the 0.7 mm DNP probe has been studied before, and it was found that the temperature varies by 10 K ranging from 95-105 K for a MAS rate change from 10 to 65 kHz.^35^ Hence, the effect of temperature on the enhancements measured in these experiments is expected to be negligible here.

The numerical simulations of relayed DNP were performed on an Apple MacBOOK Pro (2018) running Matlab R2021b with 4 cores (2.3 GHz Quad-Core Intel Core i5) and 16 GB RAM. In all the simulations, values of the analyte domain size (diameter), *L* = 0.5 μm, and the ^1^H static spin-diffusion coefficient estimated for l-Histidine·HCl·H_2_O of *D*_0,histidine_ = 0.75. 10^–3^ μm^2^ s^–1^ were used, such that the spin-diffusion coefficient at a given MAS rate is described by *D*_histidine_ = *D*_0,histidine_/MAS rate (in kHz). For simulations corresponding to standard experiments, *T*_B,source_ = 2.8 s (20 kHz) and 5.3 s (60 kHz) (experimentally obtained build-up time in the polarization source at 100 K), ε_0_ = 120 (20 kHz) and 135 (60 kHz) (experimentally obtained enhancements on TCE in the polarization source at 100 K), and *T*_1,histidine_ = 823 s (the measured spin-lattice relaxation of neat l-histidine.HCl·H_2_O), were used. As the value of the parameters *D*_histidine_, *T*_B,source_, and ε_TCE_ are MAS rate dependent, for simulations corresponding to slow-fast MAS experiments, simulations were performed over three intervals, corresponding to the value of these parameters for the MAS rate during τ_pol_–τ_up_, and two different MAS rates during τ_up_ (Figure S3 in SI). These were 37, 42, and 31 s intervals (corresponding to a total 110 s polarization time), with an approximated MAS rate of 20, 38, and 58 kHz, respectively. During the first two intervals ε_TCE_ and *T*_B,source_ values of 120 and 2.8 s were used, and during the third interval 135 and 5.3 s were used.

## Results and Discussion

### Relayed DNP Experiments

l-Histidine·HCl·H_2_O impregnated with 32 mM HyTEK-2 in TCE was used for the experiments here as this has been shown to be a good model sample for relayed DNP experiments.^12,15^ T_B,TCE_, the TCE build-up time in the radical solution at 100 K, and ε_TCE_, the enhancement on TCE, were measured using the ^1^H DEPTH NMR experiment for background suppression, in the 20 to 60 kHz MAS range, and the values are shown in Table 1. Both, the measured T_B,TCE_ and ε_TCE_ values increase with faster MAS rate, from 2.8 s at 20 kHz to 5.3 s at 60 kHz, and from 100 at 20 kHz to 135 at 60 kHz, respectively, which is consistent with previous reports and the source-sink model proposed for HyTEK-2.^25,36^

**Table 1:** Measured T_B,TCE_, ε_TCE_, and *T*_opt,his_ (on Peak Ha) of l-Histidine·HCl·H_2_O Impregnated with 32 mM HyTEK-2 in TCE as Function of the MAS Rate at 100 K

MAS rate (kHz)	*T*_B,TCE_ (s)	ε_TCE_	*T*_opt, his_ (s)
20	2.8	100 ± 8^a^	63^c^
30	3.4	122^b^	71^d^
40	3.9	136^b^	88^d^
50	4.5	137^b^	101^d^
60	5.3	124 ± 12^a^/135^b^	110^c^

a Measured using ^1^H-^13^C CPMAS NMR.

b Measured using ^1^H DEPTH NMR.

c Measured using saturation recovery ^1^H spin-lock DEPTH NMR.

d Measured using saturation recovery ^1^H–^13^C CPMAS NMR.

To confirm that the build-up of polarization was consistent with the dynamics of relayed DNP, we recorded the ^1^H NMR polarization build-up curve of l-Histidine·HCl·H_2_O, at 20 and 60 kHz MAS, in the presence and absence of microwave irradiation. As expected, from Figure S4, it can be seen that the ^1^H enhancement changes as a function of build-up time for the initial build-up time points, which is a signature of the relayed DNP.^14−16^ Again as expected, the build-up of enhancement is faster at 20 kHz compared to 60 kHz, due to faster spin-diffusion at 20 kHz MAS compared to 60 kHz. ε_∞_, the steady state enhancement, detected on the ^1^H is found to be 87 at 20 kHz and 60 at 60 kHz. This is in line with the observations made by Berruyer et al., where it was found that for relayed DNP at fast MAS, ε_∞_ decreases at faster MAS rates, and also builds-up relatively slower.^12^ As described above, relayed DNP involves the transfer of polarization from the polarizing radical solution to the bulk of the material by spin-diffusion. With faster MAS rates the ^1^H-^1^H dipolar couplings that drive spin-diffusion are averaged more efficiently, and hence faster spinning leads to lower enhancements.

### Optimal Polarization Time

For a given MAS rate, to determine the polarization build-up time with the highest signal sensitivity, which we dub *T*_opt_, the ^1^H signal intensity of l-histidine·HCl·H_2_O as a function of polarization time, τ_pol_, recorded with microwave irradiation, was divided by the square root of τ_pol_ and the maxima of this curve corresponds to the optimum τ_pol_ for a given MAS rate (the corresponding plot recorded at 60 kHz MAS rate is shown in Figure S6 in SI), and the values are given in Table 1.

We note that *T*_opt,his_ increases as a function of MAS rate from 63 s at 20 kHz to 110 s at 60 kHz. This is due to the lengthening of the build-up time, due to slower spin diffusion at higher MAS rates.

A way to increase hyperpolarization inside the analyte at faster MAS rates is to take advantage of the larger polarization build-up at slower MAS rates, i.e., allowing the buildup of polarization at slow MAS rates, and switching to fast MAS rates before detection (Figure 1a). Thus, the MAS rate change experiments were performed at *T*_opt,his_, the time point with maximum sensitivity optimized for the spinning rate used for acquisition, to further improve the sensitivity of the impregnated l-histidine·HCl·H_2_O.

### Slow-Fast MAS Experiments with Polarization Build-Up at 20 kHz

Slow-fast MAS experiments were performed to allow polarization to build-up at ν_pol_ and the MAS rate was then changed to ν_acq_ prior to acquisition, such that ν_acq_ > ν_pol_. Thus, a series of experiments were performed with polarization build-up at ν_pol_ of 20 kHz and acquisition at ν_acq_ corresponding to MAS rates of 30, 40, 50, and 60 kHz. Polarization was allowed to build-up at MAS rate ν_pol_ for time *T*_opt,his_ – τ_up_, where τ_up_ is the time taken to switch the MAS rate from ν_pol_ to ν_acq_ (Figure 1a). We note that ideally τ_up_ should be more or less instantaneous (or at least very short compared to τ_pol_), but that with current hardware on our probes is on the order of 30-60 s, and we note that this factor currently restricts the usage of this method to systems with *T*_opt,his_ comparable to or greater than τ_up_. The values of the enhancements, *T*_opt,his_, and τ_up_ measured from these experiments are reported in Figure 1c.

It can be noted in Figure 1c that for the standard ^1^H DNP MAS NMR experiment (with no MAS rate change), the steady-state enhancement monotonically decreases with faster MAS rates, with values decreasing by a factor ∼1.5 from 20 to 60 kHz MAS rates (blue bars), consistent with the slower rate of spin-diffusion at faster MAS rates. In comparison with the slow-fast MAS experiment with polarization build-up at *ν*_pol_ at 20 kHz and acquisition at *ν*_acq_, it can be seen that indeed, the slow-fast MAS experiment consistently leads to a higher enhancement, such that ε′_his,20-νacq_ increases with increasing *ν*_acq_ MAS rates. Furthermore, the maximum enhancement is achieved for the slow-fast MAS experiment with *ν*_pol_ at 20 kHz and *ν*_acq_ at 60 kHz, with an ε′_his,20–60 kHz_ of 84, compared to ε_his,20 kHz_ and ε_his,60 kHz_ of 89 and 62, respectively. It can be noted that the enhancement attained with the slow-fast MAS experiment, here, is slightly smaller than ε_his,20 kHz_, likely due to the finite time the sample spins at MAS rates faster than 20 kHz during *τ*_up_. With a shorter duration of *τ*_up_, a relatively higher overall enhancement value, close to ε_his,20 kHz_, can be attained.

### Slow-fast MAS Experiments with Acquisition at 60 kHz

To validate the ν_pol_ that leads to a maximum polarization at ν_acq_ of 60 kHz (close to the maximum achievable MAS rate at 100 K in the 0.7 mm probe), slow-fast MAS experiments were performed with ν_pol_ between 20 to 60 kHz. Here, all experiments were performed at build-up times corresponding to *T*_opt,his_, found to be 110 s at 60 kHz MAS rate. Thus, during the experiment, the time the rotor spins at ν_pol_ is 110–τ_up_. The recorded spectra are shown in Figure 1b, together with a standard experiment at 60 kHz MAS rate recorded with and without microwave irradiation. It can be seen from these spectra that MAS rate changes with larger difference between ν_pol_ and ν_acq_ led to higher enhancements. Figure 1d shows the comparison of ^1^H DNP NMR enhancements for l-histidine·HCl·H_2_O obtained from standard (blue bar) and slow-fast MAS (red bars) experiments. Compared to an enhancement of 62 at 60 kHz for the standard experiment, slow-fast MAS experiment always leads to a higher enhancement with acquisition at 60 kHz. The enhancement in the slow-fast MAS experiment goes up incrementally from 73 to 84 as ν_pol_ decreases from 50 to 20 kHz. The spheres shown above these plots schematically represent the relative extent of penetration of polarization into the l-histidine·HCl·H_2_O particles across the various slow-fast MAS experiments, showing the increased transfer of polarization into the l-histidine·HCl·H_2_O particles with polarization at slower speeds. ε_his,νpol-νacq_ were also determined for ν_pol_ at 20, 30, and 40 kHz, and the values are listed in the Table S2 in SI.

### Calculating Signal Enhancement and Polarization from Numerical Simulation of Relayed DNP

The transfer of polarization from the polarizing phase into the bulk of sample, can be simulated using numerical models previously developed in detail by Van Der Wel et al.,^37^ Rossini et al.,^14^ Pinon et al.,^15^ Prisco et al.,^16^ and subsequently extended by Berruyer et al. to the case of the fast MAS regime (up to 65 kHz).^12^ Thus, the transfer of polarization from the polarizing phase into the impregnated spherical particles is described by^12,14−16,37^
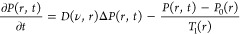
1where *P*(*r*,*t*) corresponds to the polarization at position *r* and time *t* in the system such that *r* is the distance from the center of the analyte to the considered position; *D*(ν,*r*) is the ^1^H spin diffusion coefficient that depends inversely on ν, the MAS rate,^12,19,20^ and is dependent on the ^1^H concentration in a given phase and can thus change with r from one phase to the other. *P*_0_(*r*), the local equilibrium polarization in the absence of ^1^H spin diffusion, under microwave irradiation, and *T*_1_(*r*), the ^1^H spin–lattice relaxation time, as described previously, are different in different media (i.e the polarizing phase or analyte phase) and change at the border between the two, with a hyperbolic tangent function.

Simulations were performed with inputs obtained from the slow-fast MAS experiments, and the values of the parameters are given in the Experimental Section. For simplicity, these simulations involve three segments in time, corresponding to the value of *D* for the MAS rate during τ_pol_–τ_up_, a second *D* to capture the average rate during the first half of τ_up_ (where the rate changes relatively rapidly in the experiment), and a third *D* to capture the average rate in the second half of τ_up_ (where the rate changes relatively slowly in the experiment), and where *D* has an inverse MAS rate dependence. The two different segments in τ_up_ are just a simple approximation to capture the experimental MAS rate change profile, shown in Figure 1a and SI, Figure S3.

**Figure 2:**
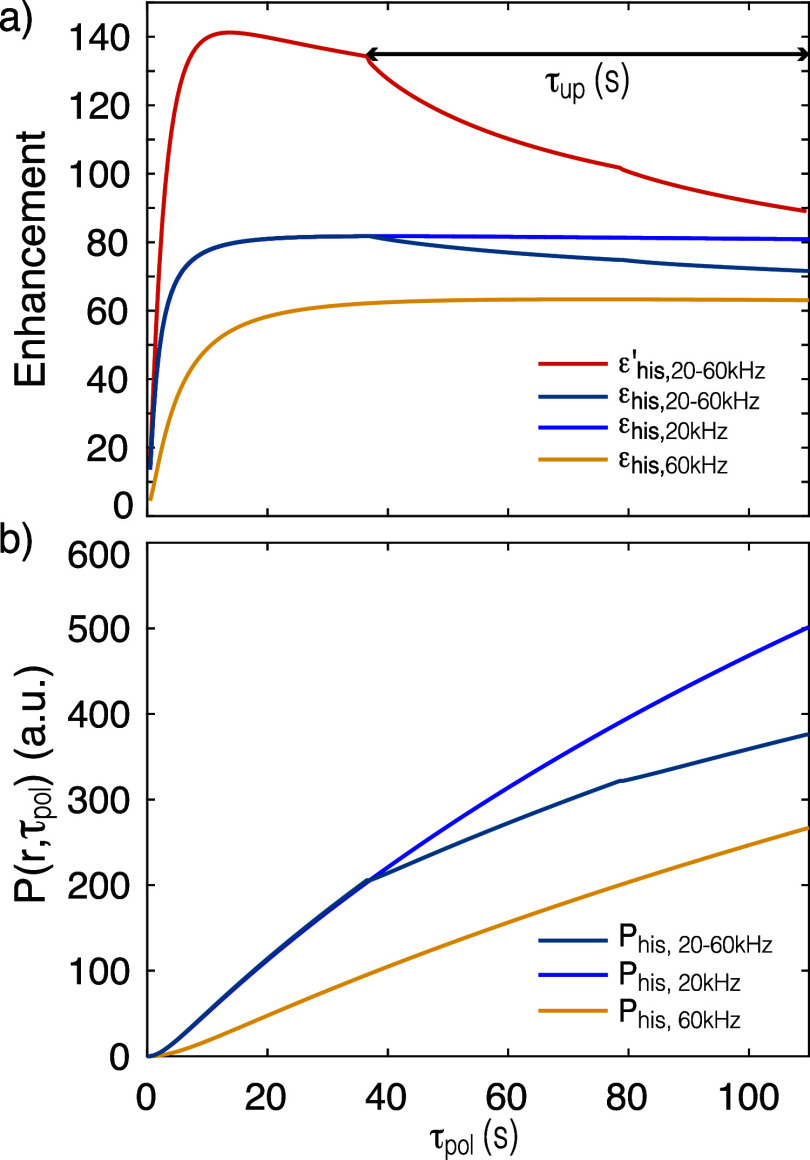
Numerical simulations of the build-up of (a) enhancement ε′_his,νpol-νacq_ and ε_his,νpol-νacq_ with ν_pol_ = 20 kHz and ν_acq_ = 60 kHz corresponding to slow-fast MAS, and ε_his,20 kHz_ and ε_his,60 kHz_ corresponding to standard DNP experiments; (b) polarization *P*_his,νpol-νacq_ with microwave irradiation, and ν_pol_ = 20 kHz and ν_acq_ = 60 kHz corresponding to slow-fast MAS, and *P*_his,20 kHz_ and *P*_his,60 kHz_ corresponding to standard DNP experiments.

Figure 2 shows the simulated enhancement, and polarization as a function of the polarization time τ_pol_, for the 20-60 kHz slow-fast MAS experiment and at 20 and 60 kHz MAS rate, for the standard experiment. Figure 2a shows the buildup of ε_his,20–60 kHz_ with polarization time and as expected until τ_up_, the buildup of polarization follows the same dynamics as that of ε_his,20 kHz_. During, τ_up_, a decrease in enhancement is observed, due to the slowing down of spin diffusion as we increase spinning rates, and the final ε_his,20–60 kHz_ = 72 at τ_pol_ = 110s (corresponding to the time point of acquisition in the experiments) is close to the experimental ε_his,20–60 kHz_ of 63. In contrast, at the same build-up time, the simulated and experimental ε′_his,20–60 kHz_ have much higher values of 89 and 84, respectively. This increase is observed because, instead of dividing the microwave on signal with the off corresponding to the same MAS rate, here it is divided by the off spectrum for the target MAS rate (60 kHz here) for the ε′_his,νpol-νacq_ measurement. As described above, ε_his,20–60 kHz_ is calculated by taking the ratio of polarization obtained from 20 to 60 kHz slow-fast MAS simulation with and without microwave irradiation. Whereas the ε′_his,20–60 kHz_ calculation differs in the microwave off polarization, which is obtained from a constant 60 kHz MAS rate simulation. Since at 110 s, the microwave off polarization of the 20 to 60 kHz slow-fast MAS simulation is higher than the 60 kHz simulation, shown in SI Figure S7, hence ε′_his,20–60 kHz_ is greater than ε_his,20–60 kHz_, consistent with the experimental observations. Similarly, Figure 2b shows that the polarization up to τ_up_ builds up at the same rate as at 20 kHz, and then slows down during τ_up_, again consistent with the slowing down of spin-diffusion at these faster MAS rates.

**Figure 3:**
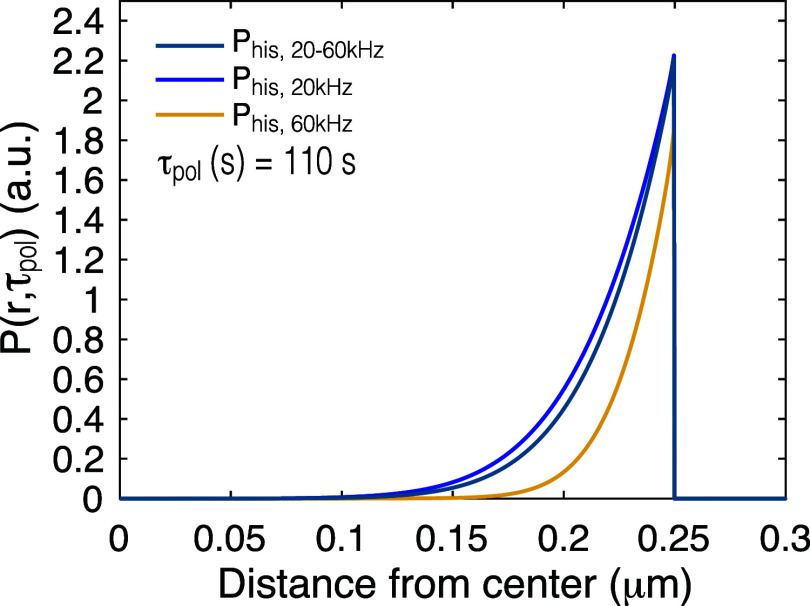
Simulated distribution of polarization inside a particle of domain size (diameter), *L* = 0.5 μm, as a function of the distance from the grain center, simulated at the build-up time τ_pol_ = *T*_opt,his_ = 110 s determined for a MAS rate ν_acq_ = 60 kHz, for 20-60 kHz slow-fast MAS (*P*_his, 20–60 kHz_), and standard experiments at 20 kHz (*P*_his, 20 kHz_) and 60 kHz (*P*_his, 60 kHz_) MAS rates.

Figure 3 shows the distribution of polarization as a function of depth inside the target particle at MAS rates 20 kHz, 60 kHz, and for a slow-fast MAS experiment with ν_pol_ = 20 kHz, and ν_acq_ = 60 kHz, calculated at τ_pol_ = 110 s. It can be seen that a faster rate of spin diffusion leads to deeper penetration of polarization, as the polarization penetrates the deepest for the case of 20 kHz, followed by the slow-fast MAS experiment, and the least for the 60 kHz MAS.

### Overall Sensitivity

**Figure 4:**
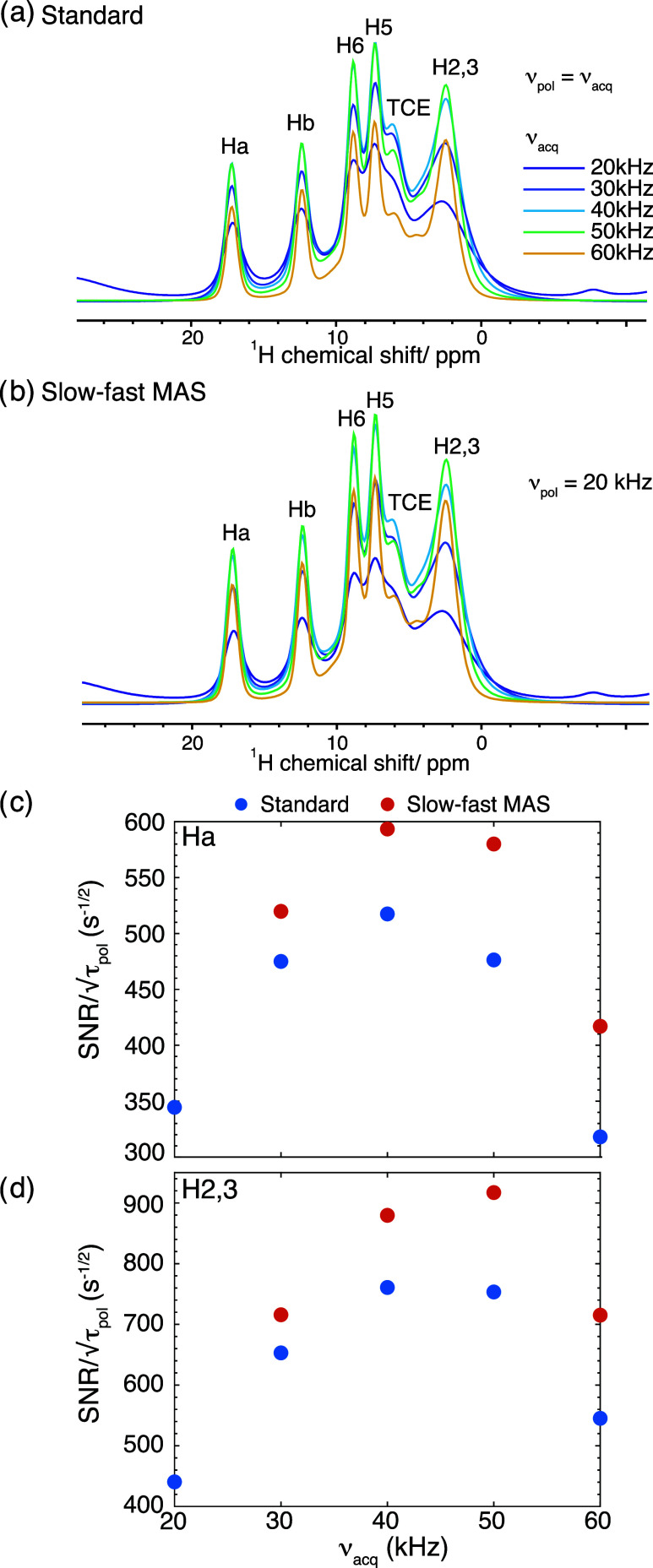
^1^H DNP MAS NMR spectra of l-histidine·HCl·H_2_O impregnated with 32 mM HyTEK-2 in TCE, recorded at 100 K with microwave irradiation at MAS rates of 20 up to 60 kHz, with DEPTH, and TCE solvent suppression, and τ_pol_ = *T*_opt,his_ determined at MAS rate ν_acq_, under (a) standard experiment with ν_pol_ = ν_acq_, and (b) with slow-fast MAS experiment with ν_pol_ = 20 kHz, and ν_acq_ as per the color scheme shown on the figure. The sensitivity at ν_acq_ for standard and slow-fast experiments obtained from these spectra, for peaks assigned as (c) Ha and (d) H2,3.

Up to now we have shown a comparison between the DNP enhancement of slow-fast and standard DNP experiments. However, the DNP enhancement alone is not sufficient to describe the overall sensitivity in 1D ^1^H detected relayed DNP experiments at fast MAS. Increasing spinning rates lead to higher ^1^H NMR detection sensitivity due to the narrowing of ^1^H line-width, and incorporation of sideband intensity into the centerband. Thus, a comparison of sensitivity between DNP-enhanced ^1^H NMR spectra acquired at different MAS rates requires an analysis of the signal-to-noise per unit square root of polarization time. Figure 4a,b show the ^1^H DNP MAS NMR spectra of l-histidine·HCl·H_2_O impregnated with 32 mM HyTEK-2 in TCE recorded at MAS rate ν_acq_ with standard (ν_pol_ = ν_acq_) and slow-fast MAS NMR experiment (ν_pol_ = 20 kHz). It can be seen in Figure 4a,b, that with faster MAS rates there is a significant improvement in ^1^H resolution due to narrowing of ^1^H line-width, as expected. Figure 4c,d shows the relative sensitivity (as opposed to the enhancement shown above) measured from the signal-to-noise per √τ_pol_, for the standard experiments and slow-fast MAS experiments at ν_pol_ = 20 kHz, as a function of ν_acq_ for Ha and H2,3 (both plots were normalized to the maxima in the plot obtained for H2,3). We have previously shown that sensitivity is site-specific, and here changes differently due to different MAS rate dependence of the line-width of these peaks. Hence, in the standard experiments, the sensitivity measured at *T*_opt,his_ (values given in Table 1) is found to be maximum at 40 kHz for Ha, and at between 40 and 50 kHz for peak H2,3, as seen previously.^13^ For the slow-fast MAS experiments at ν_pol_ = 20 kHz, there is a clear relative sensitivity boost compared to the standard experiments. Similar to the trend obtained for the enhancement, maximum sensitivity gains are obtained for the 20 to 60 kHz slow-fast MAS experiments. The trend of overall sensitivity of Ha remains essentially the same in comparison to standard experiments, with a maximum between 40 and 50 kHz, but for H2,3 there is slight shift to 50 kHz MAS which emerges as the point with highest sensitivity. It must be noted, that in the analysis herein, the currently accessible values of τ_down_ (neglected in this analysis), increases the experimental time and hence leads to an overall decrease in sensitivity, as it is comparable to the polarization time required for the experiments and hence adds significantly to the experimental time. Faster MAS rate switching will be crucial for success of this method in the future.

## Conclusions

We have shown that the ^1^H sensitivity in relayed DNP experiments at fast MAS can be maximized by allowing polarization to build-up at slow MAS and with detection at fast MAS rates, in an experiment we dub slow-fast MAS. We find that for MAS rates of 30-60 kHz, the sensitivity can be improved with slow-fast MAS experiment with a ν_pol_ of 20 kHz, leading to a 35% improvement in the sensitivity of l-histidine·HCl·H_2_O compared to standard DNP experiments at 60 kHz.

We note that currently, with the hardware we are using here for this proof of concept demonstration, τ_up_ limits the applicability of the method to systems with build-up times *T*_opt,his_ comparable to or greater than τ_up_. Similarly, τ_down_ (which is neglected in the analysis above) currently increases the experimental time and hence decreases sensitivity. Faster MAS rate changes will obviate these defects in the future. Similarly, the probes used here cannot reliably spin slower than 20 kHz in coupling with rate changes, but we expect that the sensitivity could be further improved if the spinning rate during the polarization delay ν_pol_ could be reduced from 20 kHz to around 5 kHz, where *D* is expected to be maximum.

We further note that other than the use of slow-fast MAS, other strategies for dipolar recoupling during the polarization delay could also be potentially used to improve sensitivity at fast MAS rates, and this will be explored in future studies.

## Supporting Information Available

Background suppression, spin-lock amplitudes, MAS rate profiles, build-up behaviors, together with macros used for acquisition, and a link to the raw data (PDF)
