# Supplementary material for: Maximizing Relayed 1H Hyperpolarization Transfer by Slow-Fast MAS NMR Spectroscopy
Source: J Phys Chem A. 2024 Aug 9;128(33):7005–12. doi: 10.1021/acs.jpca.4c02452 (PMC11345838; doi:10.1021/acs.jpca.4c02452)
Supplement: Supplementary file 1 — jp4c02452_si_001.pdf [file jp4c02452_si_001.pdf]

Supporting Information

Maximising Relayed ^{1}H Hyperpolarization Transfer By Slow-Fast MAS NMR Spectroscopy

Saumya Badoni,[a] Pierrick Berruyer,[a] Lorenzo Niccoli,[b,c,d,e] Anne Lesage,[b] and Lyndon Emsley[a*]

[a] Institut des Sciences et Ingénierie Chimiques, Ecole Polytechnique Fédérale de Lausanne (EPFL), CH-1015 Lausanne, Switzerland

[b] Université de Lyon, Centre de Resonance Magnétique Nucléaire (CRMN) à Très Hauts Champs de Lyon (UMR 5082 - CNRS, ENS Lyon, UCB Lyon 1), 69100 Villeurbanne, France

[c] Center of Magnetic Resonance (CERM), University of Florence, 50019 Sesto Fiorentino, Italy

[d] Departement of Chemistry "Ugo Schiff", University of Florence, 50019 Sesto Fiorentino, Italy

[e] Consorzio Interuniversitaio Risonanze Magnetiche Metalloproteine (CIRMMP), 50019 Sesto Fiorentino, Italy

*corresponding author: lyndon.emsley@epfl.ch

Table of contents

1. Supplementary figures S2
2. Supplementary tables and details S6

Raw data statement

The NMR raw data is available at https://zenodo.org/doi/10.5281/zenodo.13222521 in the original TopSpin, JCAMP formats, along with other data related to this work. Data are made available under the license CC-BY-4.0 (http://creativecommons.org/licenses/by/4.0/ Creative Commons Attribution 4.0 International).

1. Supplementary figures

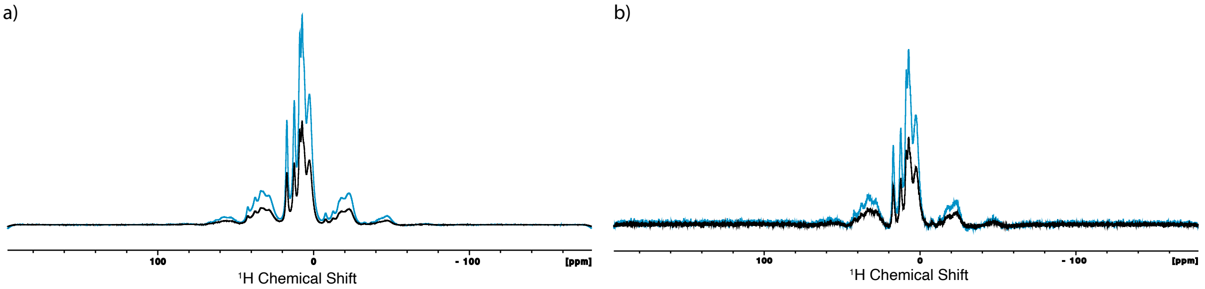

Figure S1. ¹H DNP MAS NMR spectra of L-histidine·HCl·H_2O impregnated with 32 mM HyTEK-2 in TCE, recorded at MAS rate of 20 kHz, with DEPTH, and TCE solvent suppression, and τ_{pol} = 10 s, recorded at 100 K a) with microwave irradiation, and b) without microwave irradiation, with 8 (spectra in black) and 16 scan (spectra in blue) phase cycling of DEPTH sequence. Clearly, from this figure it can be seen that 8 scan phase cycling is sufficient to perform background suppression and obtaining a flat baseline.

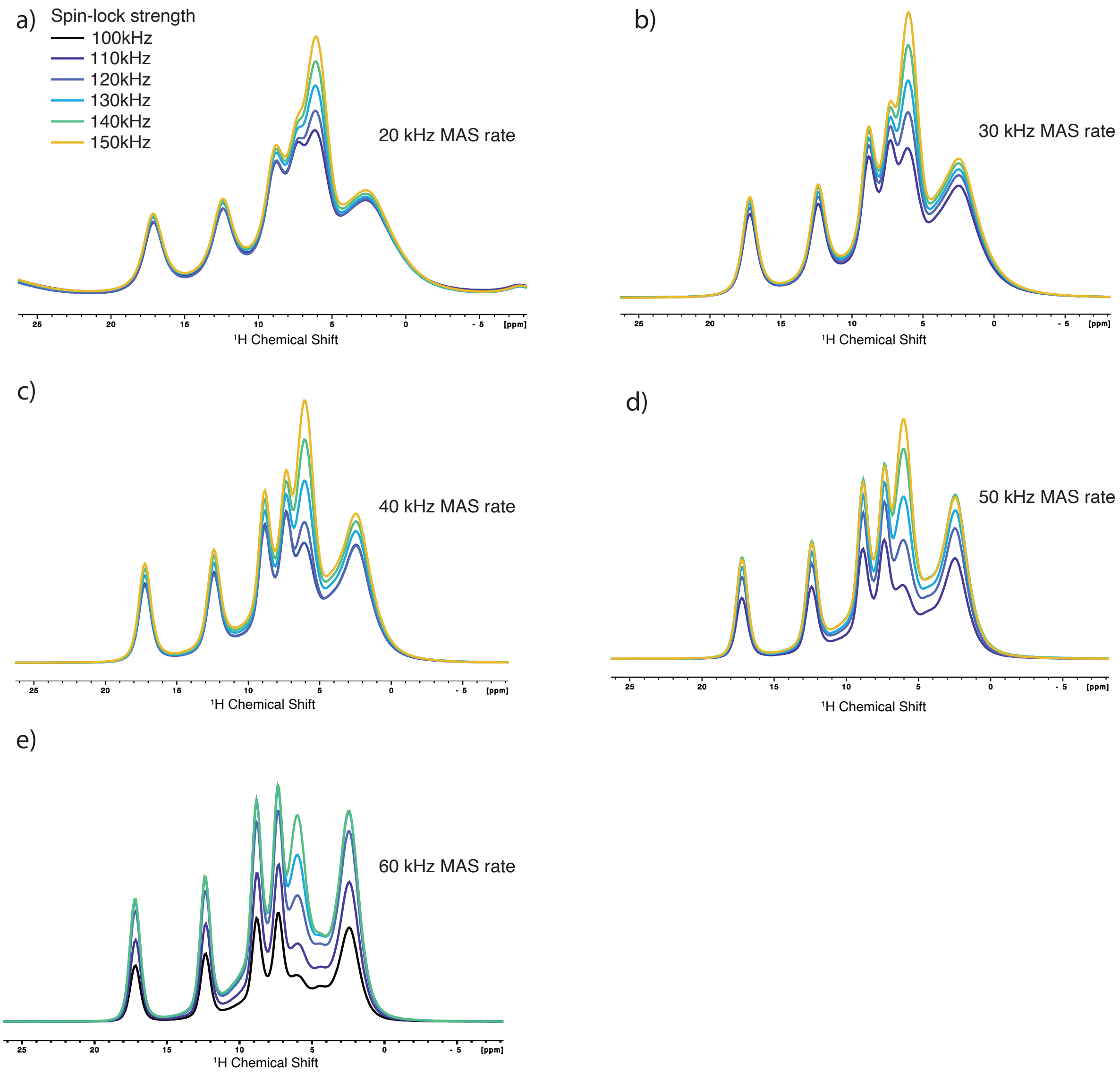

Figure S2. ¹H DNP MAS NMR spectra of L-histidine·HCl·H_2O impregnated with 32 mM HyTEK-2 in TCE, recorded at MAS rates of a) 20, b) 30, c) 40, d) 50, and e) 60 kHz, with DEPTH, and TCE solvent suppression, recorded at 100 K with microwave irradiation, with all the same parameters at a given MAS rate and varying the spin-lock strength for optimization. Subsequently, a spin-lock strength of 130,150, 150, 140, and 130 kHz was chosen, for MAS rates 20, 30, 40, 50, and 60 kHz, respectively.

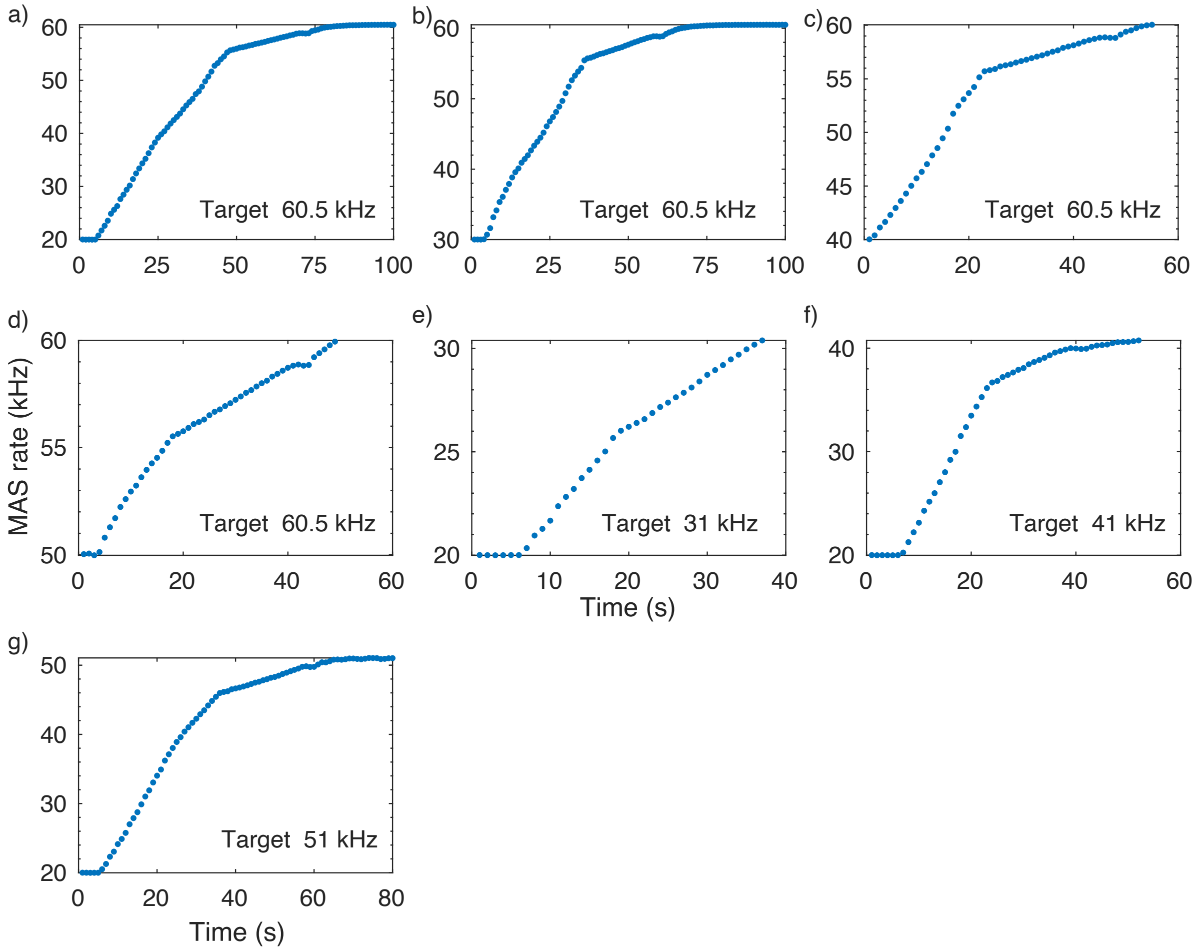

Figure S3. MAS rate change profile for the slow-fast MAS experiments with ν_{pol}= a) 20, b) 30, c) 40, d) 50 kHz and ν_{acq}=60kHz; and ν_{pol}= 20 kHz and ν_{acq}= e) 30, f) 40, g) 50 kHz along with the MAS rate target setpoint given on the plots.

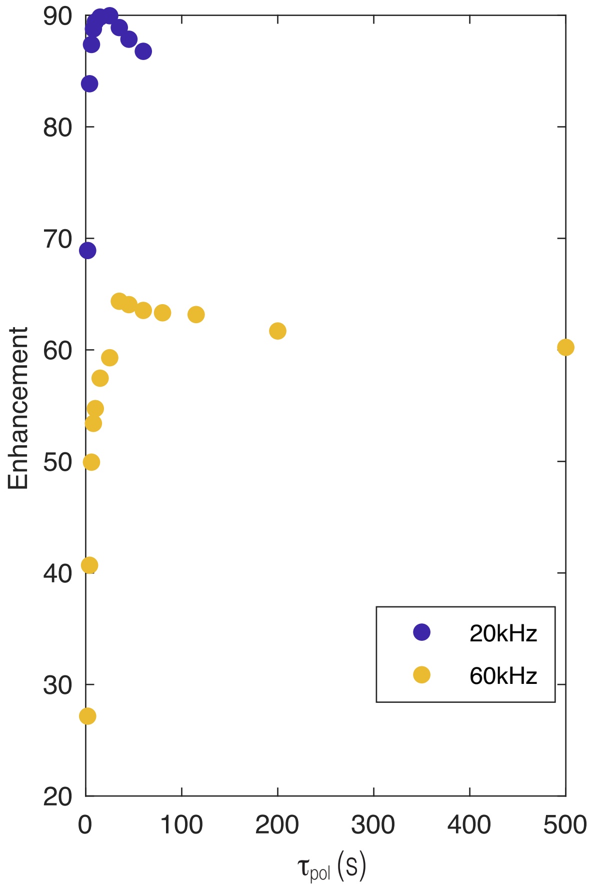

Figure S4. DNP enhancement measured from ^{1}H NMR signal integral (60 kHz MAS rate)/intensity (20 kHz MAS rate) as a function of polarization time, τ_{pol}, under microwave irradiation for the signals assigned to Ha, of L-histidine·HCl·H_2O impregnated with 32 mM HyTEK-2 in TCE.

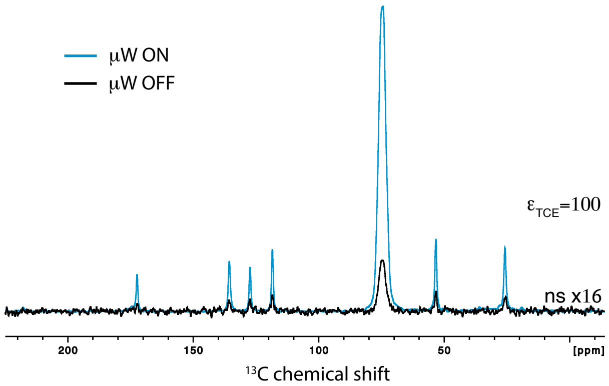

Figure S5. ^{13}C CPMAS NMR spectra of L-histidine·HCl·H_2O impregnated with 32 mM HyTEK-2 in TCE, recorded at MAS rate of 20 kHz, at the build-up time 1.3*$T_{B,TCE}$ at 20 kHz, recorded at 100 K with (blue) and without (black) microwave irradiation. The spectrum without microwave irradiation was recorded with 16 times more scans compared to the spectrum with microwave irradiation.

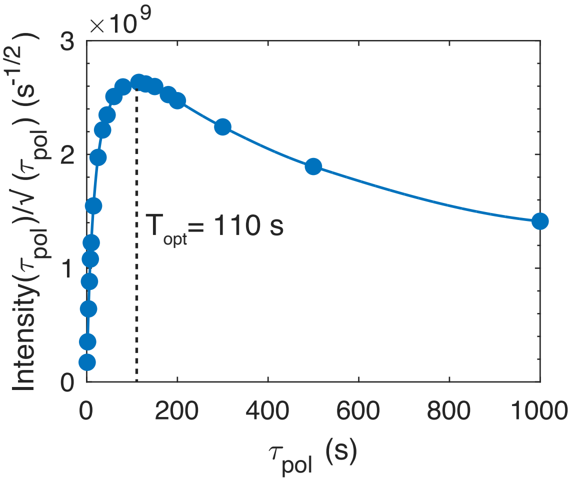

Figure S6. Build-up of ^{1}H NMR signal intensity per unit square root of polarization time, τ_{pol}, of peak Ha, obtained from ^{1}H DNP MAS NMR spectra of L-histidine·HCl·H_2O impregnated with 32 mM HyTEK-2 in TCE, recorded at 100 K with microwave irradiation at MAS rate of 60 kHz, with DEPTH, and TCE solvent suppression.

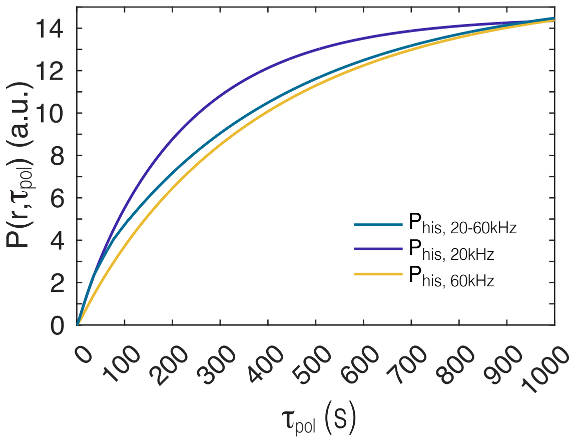

Figure S7. Simulated ^{1}H signal polarization as a function of polarization time, τ_{pol}, without microwave irradiation of L-histidine·HCl·H_2O impregnated with 32 mM HyTEK-2 in TCE for 20-60 kHz slow-fast MAS ($P_{his,\ 20\text{-}60kHz}$), and standard experiments at 20 ($P_{his,\ 20kHz}$) and 60 kHz ($P_{his,\ 60kHz}$). The parameters used for simulations are described in experimental section of the manuscript.

Supplementary tables and details

The error bar ($\Delta\varepsilon$) estimation on the ^{1}H DNP enhancement (ε) was perfomed as follows:

$$\Delta\varepsilon = \varepsilon\left(\frac{\Delta I_{ON}}{I_{ON}} + \frac{\Delta I_{OFF}}{I_{OFF}}\right) \qquad (S1)$$

here ΔI_{ON} and ΔI_{OFF} represent the error on the ^{1}H NMR signal intensity I_{ON}(with microwave irradiation) and I_{OFF} (without microwave irradiation), respectively, determined from the noise level.

MAS rate (kHz)	$T_{B,1}$ (s) (relative contribution)	$T_{B,2}$ (s) (relative contribution)
20	50 (0.86)	1007 (0.25)
30	54 (0.85)	947 (0.25)
40	68 (0.89)	946 (0.18)
50	77 (0.75)	994 (0.19)
60	83 (0.27)	1082 (0.1)

Table S1. Build-up times and their relative contribution measured on peak Ha of histidine.HCl.H_2O impregnated with 32 mM HyTEK2 in TCE at 95 to 105 K under microwave irradiation. These values were fitted and used for the determination of T_{opt}.

ν_{pol} (kHz)	ν_{acq} (kHz)	$\varepsilon_{his,\nu pol-\nu acq}$
20	60	63
30	60	67
40	60	68

Table S2. The values of $\varepsilon_{his,\nu pol-\nu acq}$ measured using slow-fast MAS experiments from ^{1}H DNP MAS NMR spectra of L-histidine·HCl·H_2O impregnated with 32 mM HyTEK-2 in TCE.

MACROS used for recording 1 scan of 20-60 kHz slow-fast MAS experiments

```
#110s buildup
import array
import string

#(experiment number as setup in topspin directory)
expsat = 8

#(experiment number as setup in topspin directory)
expno = 28

#1 more than actual number of scans
scans = 2

#1 more than actual number of scans
num_delays = 2

mas_high = 60500

delays = [109]

mas_settle_time_up = 76

for i in range(1,num_delays):
	for j in range(1,scans):
		XCMD("re " + str(expsat) + " 1", wait = WAIT_TILL_DONE)
		XCMD("zg", wait = WAIT_TILL_DONE)
		SLEEP(delays[i-1]-mas_settle_time_up)
		XCMD("masr " + str(mas_high), wait = WAIT_TILL_DONE)
		XCMD("masrset", wait = WAIT_TILL_DONE)
		SLEEP(mas_settle_time_up)
		XCMD("re " + str(expno), wait = WAIT_TILL_DONE)
		XCMD("zg", wait = WAIT_TILL_DONE)
```
